# Changes of Milk Metabolomic Profiles Resulting from a Mycotoxins-Contaminated Corn Silage Intake by Dairy Cows

**DOI:** 10.3390/metabo11080475

**Published:** 2021-07-23

**Authors:** Gabriele Rocchetti, Francesca Ghilardelli, Paolo Bonini, Luigi Lucini, Francesco Masoero, Antonio Gallo

**Affiliations:** 1 Department of Animal Science, Food and Nutrition, Faculty of Agricultural, Food and Environmental Sciences, Università Cattolica del Sacro Cuore, 29122 Piacenza, Italy; francesca.ghilardelli@unicatt.it (F.G.); francesco.masoero@unicatt.it (F.M.); antonio.gallo@unicatt.it (A.G.); 2 Department for Sustainable Food Process, Faculty of Agricultural, Food and Environmental Sciences, Università Cattolica del Sacro Cuore, 29122 Piacenza, Italy; luigi.lucini@unicatt.it; 3 oloBion—OMICS LIFE LAB, 08028 Barcelona, Spain; pb@olobion.ai

**Keywords:** milk metabolomics, mass spectrometry, multivariate statistics, mycotoxins

## Abstract

In this study, an untargeted metabolomics approach based on ultra-high-performance liquid chromatography coupled with high-resolution mass spectrometry (UHPLC-HRMS) was used for investigating changes in chemical profiles of cow milk considering diets based on mycotoxins-contaminated corn silages. For this purpose, 45 milk samples were classified into five clusters according to the corn silage contamination profile, namely (1) low levels of *Aspergillus*- and *Penicillium*-mycotoxins; (2) low levels of fumonisins and other *Fusarium*-mycotoxins; (3) high levels of *Aspergillus*-mycotoxins; (4) high levels of non-regulated *Fusarium*-mycotoxins; (5) high levels of fumonisins and their metabolites, and subsequently analyzed by UHPLC-HRMS followed by a multivariate statistical analysis (both unsupervised and supervised statistical approaches). Overall, the milk metabolomic profile highlighted potential correlations between the quality of contaminated corn silages (as part of the total mixed ration) and milk composition. Metabolomics allowed to identify 628 significant milk metabolites as affected by the five levels of corn silage contamination considered, with amino acids and peptides showing the highest metabolite set enrichment (134 compounds). Additionally, 78 metabolites were selected as the best discriminant of the prediction model built, possessing a variable importance in projection score >1.2. The average Log Fold-Change variations of the discriminant metabolites provided evidence that sphingolipids, together with purine and pyrimidine-derived metabolites were the most affected chemical classes. Also, metabolomics revealed a significant accumulation of oxidized glutathione in milk samples belonging to the silage cluster contaminated by emerging *Aspergillus* toxins, likely involved in the oxidative imbalance. These preliminary findings provide new insights into the potential role of milk metabolomics to provide chemical indicators of mycotoxins-contaminated corn silage feeding systems.

## 1. Introduction

Corn silage is one of the main ingredients characterizing the feeding systems of lactating cows in the farms of the Po Valley (North-Italy), with exception of those producing milk processed into some Protected Denomination of Origin cheeses [1]. However, corn silage can be contaminated with mycotoxins, secondary metabolites produced by several fungi, which can influence animals’ health when ingested [2,3]. The ubiquitous nature of mycotoxins and the severity of the effects of some mycotoxins on human health, because of carry over phenomena in dairy products, make them a major food safety concern. Overall, several mycotoxins are successfully inactivated by the rumen flora, whereas others pass unchanged or are converted into metabolites that retain biological activity [4]. Therefore, the barrier function of the rumen largely determines the susceptibility of dairy cows towards individual mycotoxins [5]. An impairment of this barrier function may increase the absorption rates, thus determining not only potential adverse health effects to animals, but also the excretion of mycotoxins and their biologically active metabolites into milk [4].

The occurrence of mycotoxins in silage has been comprehensively reviewed [6,7], with particular emphasis on the occurrence, effects, prevention, and mitigation strategies. Going into details, ruminants can be less severely affected by certain mycotoxins compared to monogastrics, which is attributed to the microbial activity in the rumen that can modify mycotoxins into less toxic compounds [8,9]. In the last years, several works have deepened the co-occurrence of regulated and emerging mycotoxins in corn silages, by focusing on different parameters, such as fermentation quality and bacterial communities [7,10,11]. Regarding the available literature describing cow milk as a function of the mycotoxins’ contamination of corn silage, Signorini et al. [12] developed a simulation model to carry out a risk exposure assessment of the mycotoxin level (namely aflatoxin B1, deoxynivalenol, and zearalenone) in cow’s milk produced in Argentina. The authors showed that mycotoxin levels in corn silage and concentrated feeds were the factors mostly correlated with mycotoxin concentrations in milk, thus recommending a strong monitoring plan of both silages and milk samples to reduce the risk of contamination. The importance of corn silage from a nutritional standpoint and considering the dairy cows’ performance and milk quality has been widely studied [13]. Recently, Tharangani et al. [14] developed a corn silage quality index combining milk yield together with silage nutritional and fermentation parameters. These authors provided evidence that most of the variation in milk yield and composition is strictly related to silage quality. Besides, other silage quality factors potentially influencing milk production are represented by digestible neutral detergent fiber (NDF) after 30-h in vitro incubation (g/kg NDF), and concentrations (on dry matter basis) of starch, crude protein, ether extract, ammonia, and lactic acid [14].

However, current research on mycotoxins in dairy ruminants has been basic and focused on the modifications of few biochemical parameters [15]. The analysis of only few indicators (such as serum biochemical, antioxidant, and immune indices) provides limited information and allows only simple metabolic inferences to be drawn [15]. Therefore, studies based on the changes in chemical composition of cows’ biofluids as affected by mycotoxin exposure are gaining great interest [16,17]. Interestingly, the development of -omics approaches, such as targeted and untargeted metabolomic fingerprinting, has allowed researchers to potentially study the changes of multiple biofluids composition following the exposure to different mycotoxins [15]. However, there is a scarcity of available works trying to correlate the metabolomic profile of milk to the mycotoxins intake from contaminated corn silages or other feeding systems. In this regard, a study by Wang et al. [15] evaluated the effects of aflatoxin B1 added in different concentrations to the total mixed ration (TMR) on biofluids biomarkers (examined with metabolomic and biochemical tests) and demonstrated that nine milk metabolites were significantly affected by the exposure, mainly involved in the pathway of amino acids metabolism. Regarding other works available on milk metabolomics, Klein et al. [18] have successfully detected marker compounds correlated to ketosis, while Tian et al. [19] identified markers related to heat stress of dairy cows [19]. Taken together, the studies mentioned suggested that the concentrations of metabolites in milk could reflect the dairy cows’ performance under various circumstances.

To the best of our knowledge, there is still limited comprehensive information regarding the potential of ultra-high-pressure liquid chromatography (UHPLC)-high-resolution-mass-spectrometry (HRMS)-based metabolomics to discriminate the chemical fingerprint of milk samples based on the intake of mycotoxins-contaminated corn silages. This aspect could be of great interest for dairy industry, on one side because it provides a robust analytical platform for screening chemical composition of milk [20] and, on the other side, because it allows the evaluation of the quality of the corn silage used [21]. Based on this, untargeted metabolomics based on UHPLC-Orbitrap mass spectrometry was used in this work to examine milk samples collected from dairy farms using a corn silage feeding systems. The corn silages were previously classified in five main clusters according to the mycotoxins’ contamination [10]. Therefore, metabolomics followed by multivariate statistics are expected to provide new insights into the chemical perturbations of milk metabolome, as induced by both high- and low-mycotoxins contaminated corn silages feeding systems.

## 2. Results and Discussion

### 2.1. Metabolomic Discrimination of Milk Samples According to Corn Silages’ Contamination

In this work, the cohort of visited dairy farms was typical for specific intensive dairy production systems (e.g., milk yield of 32.3 ± 4.6 kg/cow/day, DMI of 23.76 ± 2.24 kg DM/cow/day), in which the corn silage represents the main ingredient of TMR (i.e., 30.51 ± 5.84% on a DM basis), with no significant differences in the management of the herd as well as adopted nutritional strategies (e.g., Net Energy for Lactation at three times the maintenance level of 1.63 ± 0.04 Mcal/kg DM or dietary CP of 16.42 ± 1.04% DM) (Table 1).

Starting from these background conditions, we used an untargeted metabolomics analysis based on UHPLC-HRMS to analyze the sampled bulk milk samples to find potential correlations between milk metabolome and corn silage quality (as referred to the mycotoxins contamination profile presented in Table 2).

**Figure 1:**
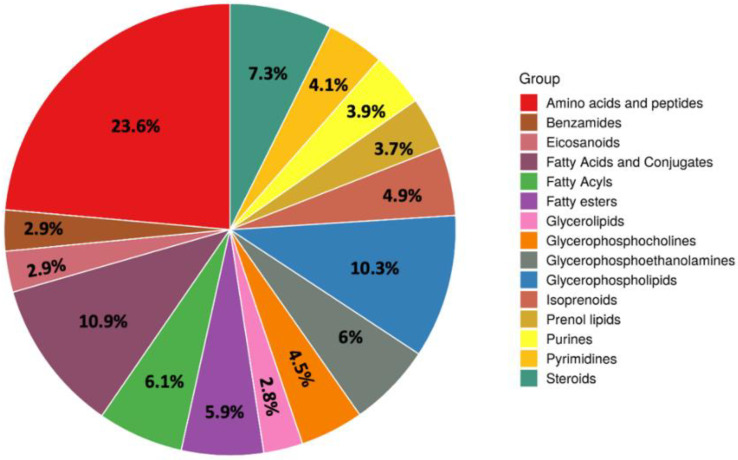
Pie chart showing the chemical classes annotated by untargeted metabolomics in the different milk samples considering those metabolite sits containing at least two entries.

The untargeted metabolomic analysis resulted in the identification of 2103 metabolites, that were annotated according to a Level 2 of confidence in annotation [20,21,22]. In our experimental conditions, a wide variety of chemical classes was found, by exploiting the recently developed and comprehensive “Bovine Metabolome Database” (BMDB) [23]. An overview of the most important chemical classes annotated was represented using the pie chart reported in Figure 1.

The high number of milk metabolites obtained by using untargeted metabolomics denotes the complexity of the food matrix under investigation. In this work, amino acids and peptides was the most numerically represented class (177 compounds), followed by several sub-classes of lipids and derivatives (such as glycerophospholipids, glycerolipids, fatty esters, fatty acyls, glycerophosphocolines, and glycerophosphoethanolamines). The nature of the untargeted metabolomics approach allowed identification of feed-derived compounds (such as plant secondary metabolites), together with purine and pyrimidine derivatives (cumulatively accounting for 60 compounds), and prenol lipids (including terpenoids). Interestingly, some fungal metabolites were detected, such as alpha-zearalenol, beauvericin, cyclopenin, and tentoxin (Table S1). Some of the fungal metabolites have been detected also in the corn silages previously analyzed by Gallo et al. [10] for their mycotoxins’ contamination profile, thus revealing potential carry-over phenomena that deserve future ad-hoc studies. Besides, the analysis of pooled quality control samples allowed to confirm the identity of 325 milk metabolites structurally also, according to spectral MS/MS marching and/or in silico annotations (Table S1). The entire list of milk metabolites annotated according to our analytical workflow is provided as Table S1, together with their MS and MS/MS spectra.

**Figure 2:**
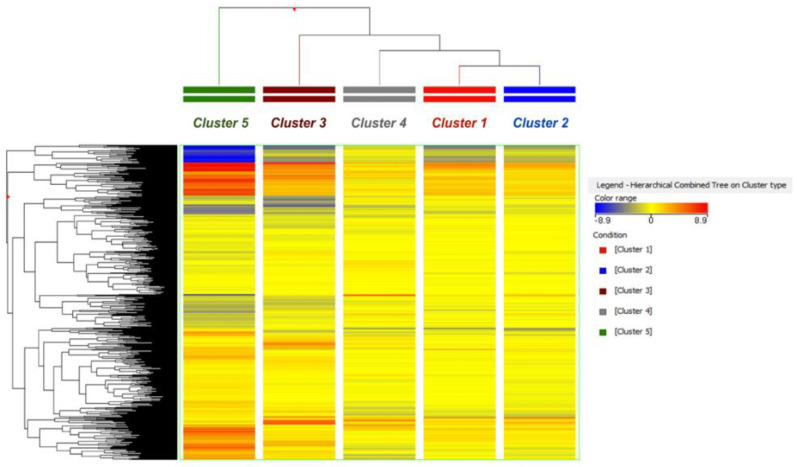
Unsupervised hierarchical clustering built considering the averaged milk metabolic profile outlined by the UHPLC-HRMS analysis.

As next step, we used the unsupervised principal component analysis (PCA) to visualize the holistic picture of the data, considering the different number of milk samples characterizing each cluster. The corresponding PCA score plot is provided in Table S1. In our experimental conditions, several variables related to both animals and dairy farm conditions contributed to the milk metabolomic profile observed, not only the contamination of corn silages by mycotoxins. This aspect was confirmed by inspecting the percentage of variability explained by two principal components (PC1 and PC2), accounting for a 15.7%, and showing only a tendency in sample grouping. Therefore, the unsupervised hierarchical clustering was used to build a heat-map (Figure 2) according to the average fold-change (FC) variations of each metabolite across the five different clusters.

Interestingly, the hierarchical clustering on milk reflected the grouping based on corn silage contamination; in fact, cluster 1 and cluster 2 (i.e., those milk samples associated to silages with low levels of mycotoxins’ contamination) were grouped together, while clusters 3, 4, and 5 showed more distinct chemical profiles. The grouping shown in Figure 2 provided evidence that corn silages contaminated by mycotoxins were able to deeply affect the chemical composition of the milk samples under investigation, thus highlighting a potential correlation between the quality of corn silage and the resulting milk composition. Also, the heat-map (Figure 2) clearly revealed that milk samples belonging to cluster 5 (i.e., silages contaminated by high levels of fumonisins and their metabolites) showed the most exclusive chemical profile, being characterized by specific up- and down-accumulated metabolites (as highlighted by the red color spots of the heat map; Figure 2) when compared to the other milk cluster types.

**Figure 3:**
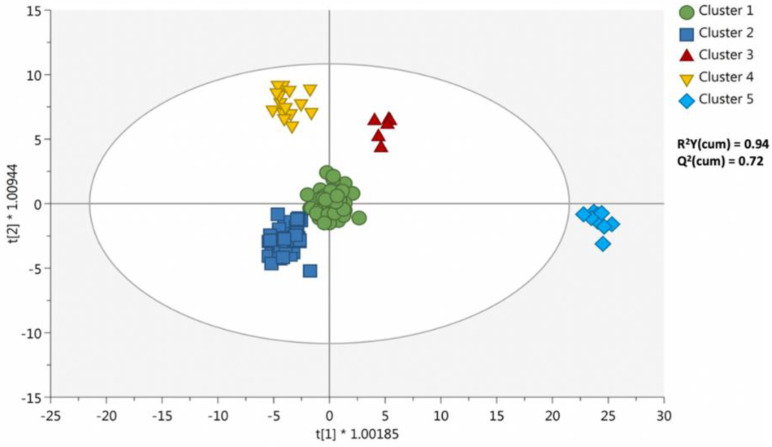
Orthogonal projection to latent structures (OPLS) discriminant analysis (DA) considering the cluster-type as class discrimination parameter.

Thereafter, the supervised OPLS-DA approach was used to extrapolate the most discriminant compounds, i.e., those milk metabolites driving the discrimination according to the cluster-type. Accordingly, this supervised method can find potential association between covariates and response variables, thus providing accurate degree of predictions. OPLS-DA is widely used in metabolomics research for identifying those biomarker compounds maximizing group separations [20]. The predictive OPLS-DA score plot is provided as Figure 3.

As can be observed, the score plot allowed to confirm the output provided by the hierarchical clustering heat map. Indeed, cluster 1 and 2 were very closed into the score plot, while the other milk samples showed completely different profiles. In addition, the OPLS-DA model possessed more than acceptable validation parameters, being the goodness-of-fit (R^2^Y) = 0.94 and the goodness-of-prediction (Q^2^) = 0.72. Besides, the permutation plot (number of random permutations = 100) allowed to exclude the model overfitting, while cross validation (CV)-ANOVA provided a *p*-value < 0.001 (Table S1).

Regarding the discriminant metabolites extrapolated by VIP selection method, 485 milk metabolites showed a VIP score >0.8 (i.e., good prediction ability). The VIP selection method outlined that the group composed by amino acids and peptides accounted for the highest number of discriminant compounds (113), followed by lipids (including fatty acyls, glycerolipids, glycerophospholipids, and sphingolipids), and other classes of compounds (Table S1). Interestingly, 78 compounds were selected as the best discriminant ones, being characterized by a VIP score >1.2. Interestingly, the highest VIP scores were recorded for five milk metabolites, namely chitobiose (1.97), 4-Hydroxy-5-(3′,4′-dihydroxyphenyl)-valeric acid-*O*-glucuronide (1.89), indoleacetic acid (1.79), *O*-succinyl-L-homoserine (1.78), and 1-hydroxypyrene glucuronide (1.73). Regarding those compounds possessing the highest prediction scores, chitobiose, belongs to the class of organic compounds known as acylaminosugars. In cattle, chitobiose is involved in the metabolic pathway called the amino sugar metabolism pathway. Besides, 4-Hydroxy-5-(3′,4′-dihydroxyphenyl)-valeric acid-*O*-glucuronide belongs to the class of organic compounds known as *O*-glucuronides; according to the BMDB [23], this compound is considered a waste product of liver and/or kidney metabolism in cattle. The VIP marker *O*-succinyl-L-homoserine (included in the cysteine and methionine metabolism) is a derivative of L-homoserine and it has a role as a both yeast and bacterial metabolite [24]. The compound indoleacetic acid belongs to the class of indoles and derivatives; it was previously reported by Zhang and co-authors [25] as a discriminant metabolite in the ruminal fluid of high-yield and low-yield group of dairy cows in terms of milk production. Finally, 1-hydroxypyrene glucuronide has been previously described in milk as a bioindicator of polycyclic aromatic hydrocarbon exposure of ruminants [26]. Pyrene is the parent compound that undergoes simple metabolism to 1-hydroxypyrene to form 1-hydroxypyrene glucuronide excreted in milk and urine [26].

### 2.2. Pathway Analysis and Significant Changes of Milk Metabolites

**Figure 4:**
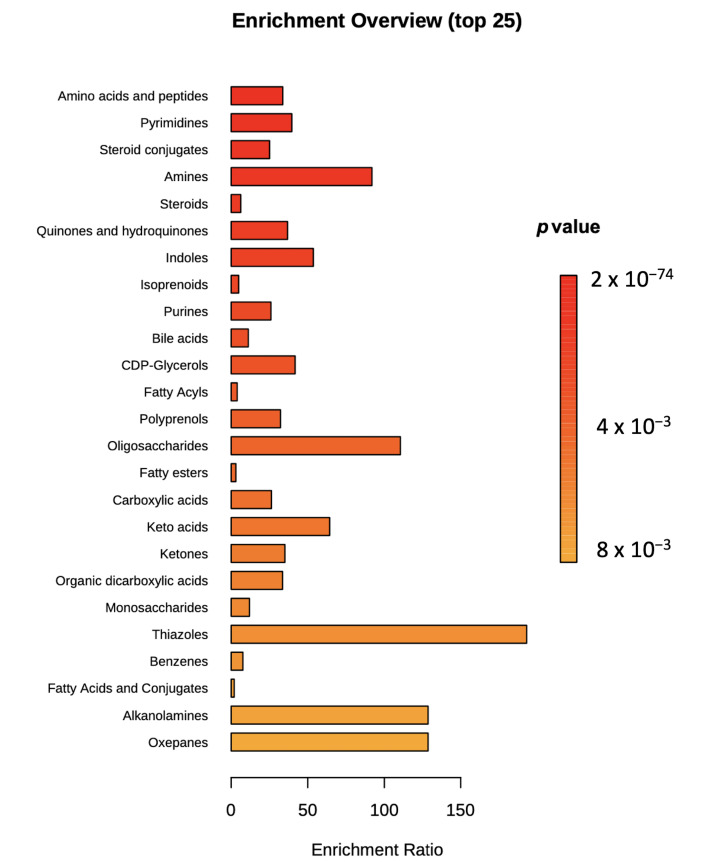
Significant metabolite set enrichment graph considering those milk metabolites annotated by UHPLC-HRMS and passing one-way ANOVA (*p* < 0.05).

As shown in Figure S1, the metabolome view map built according to the milk metabolites annotated by UHPLC-HRMS revealed that the most represented metabolic pathways were those of vitamin B6 metabolism, sphingolipid metabolism, cysteine and methionine metabolism, pyrimidine metabolism, taurine and hypotaurine metabolism, and folate metabolism. Also, other pathways presenting a lower degree of impact according to the annotated milk metabolites were those involving amino acids, such as glutamine and glutamate metabolism, and phenylalanine, tyrosine, and tryptophan biosynthesis (not shown). Considering the high number of information enclosed in the metabolomic dataset, we used one-way ANOVA (*p* < 0.05) combined with a multiple testing correction (i.e., Bonferroni Family-Wise Error Rate) to reduce the data complexity, thus obtaining 628 significant milk metabolites potentially changing according to the intake of corn silages contaminated by mycotoxins. Thereafter, these metabolites were classified, and a VIP score was also assigned according to the OPLS-DA prediction model built. Additionally, a metabolite set enrichment approach was used to assess those chemical classes most explained by the milk metabolites annotated by UHPLC-HRMS. The significant classes of milk metabolites showing the best chemical enrichment are reported as Figure 4.

In our experimental conditions, 17 classes showed a significant enrichment (*p* < 0.05); in particular, amino acids and peptides were the most represented class of compounds, followed by pyrimidines and steroid conjugates. The significant metabolites composing each enriched class are reported in Table S1.

As next step, to compare the average variation of the main classes between the milk samples and according to the different contamination of corn silages, a Volcano-Plot analysis was done considering those significant milk metabolites outlined by the different statistical approaches. Table 3 reports the average Log Fold-Change (FC) variations for each comparison against cluster 1 and cluster 2 (i.e., low-contaminated corn silages group) together with the most discriminant compound for each superclass.

As can be observed from Table 3, those milk samples associated to a contamination cluster were characterized by exclusive averaged LogFC variations. According to the most represented pathways outlined in Figure S1, we found an overall up-accumulation of sphingolipids when comparing clusters 3, 4, and 5 with the clusters 1 and 2. Also, milks samples characterizing clusters 3 and 5 showed significant up-accumulation values for pyrimidine nucleotides. Regarding the class of amino acids and peptides (i.e., the class consisting in the highest number of discriminant metabolites; Table S1), we detected averaged up-accumulation values for clusters 3 and 5, while cluster 4 showed slight down-accumulation trends. Taken together, the average LogFC variations provided evidence that sphingolipids, together with purine and pyrimidine derivatives were the most affected chemical classes and potentially related to the intake of contaminated corn silages. Interestingly, the untargeted metabolomics-based approach showed also significant variations for some plant-derived compounds, such as polyphenols, alkaloids, and prenol lipids (Table 3), as previously reported in our previous work [21], thus outlining that corn silage (as part of the feeding system) could affect the metabolomic profile of bulk milk, with lipids and polyphenols representing potential discriminant biomarkers.

Among the 134 metabolites characterizing the class of amino acids and peptides, two compounds were found to possess the highest discrimination potential, namely *O*-Succinyl-l-homoserine (VIP score = 1.78) and glycyl-glycine (VIP score = 1.68), both particularly abundant in milk samples belonging to cluster 4 (averaged LogFC values of 1.60 and 0.80, respectively; Table S1). In a previous work by Sun et al. [16], potential biomarkers for milk quality were proposed by studying the metabolomic profile of four biofluids from dairy cows. Overall, the authors reported that eight milk metabolites belonging to amino acids and peptides were significantly affected by the forage quality (i.e., high- vs low-quality forages), mainly involved in the Gly, Ser, and Thr metabolism. Regarding other discriminant classes, several compounds belonging to pyrimidine derivatives were found as discriminant, including imidazopyrimidines (such as hypoxanthine, possessing a VIP score = 1.04), nucleotides (such as dUMP, VIP score = 1.29), and nucleosides (e.g., thymidine, VIP score = 1.09). These discriminant classes (such as purine or pyrimidine derivatives) outlined by the VIP selection method could be associated to microbial protein synthesis in the rumen. In fact, as suggested by Sun et al. [16], milk protein secretion in dairy cows is strictly associated with the supply of metabolizable protein that is derived from microbial protein and undegraded dietary protein in the rumen. Additionally, in this work, the group composed by steroid and derivatives consisted in 43 milk metabolites, with the highest discriminant potential recorded for vitamin D2 3-glucuronide. In this regard, a strong down-accumulation of this metabolite was outlined for clusters 3 and 5 when compared to the cluster 1 and 2, showing averaged LogFC values of −3.27 and −5.03, respectively. On the other hand, milk samples belonging to cluster 4 (i.e., high levels of non-regulated *Fusarium*-mycotoxins) showed a significant increase for this metabolite (averaged LogFC = 1.02). Overall, cattle naturally acquire vitamin D as vitamin D2 (from plant-associated fungi) or as vitamin D3 (synthesized endogenously in sun-exposed skin from 7-dehydrocholesterol). In this regard, cattle are reported to acquire appreciable amount of vitamin D2 from corn silage (containing approximately 500 IU of vitamin D2/kg of DM) [27]; therefore, the significant down-accumulation of vitamin D2 3-glucuronide detected for clusters 3 and 5 could be an indirect parameter of malabsorption syndromes caused by the intake of mycotoxins when considered fat soluble vitamins, although further studies are mandatory [28].

Another important aspect that should be explored and deserves future ad-hoc investigations is the possible induction of oxidative stress and generation of reactive oxygen species, which is recognized as one of the most important imbalances caused by mycotoxins’ contamination. In fact, the imbalance between free radicals generated by mycotoxins and the antioxidant defense system is reported to cause a cascade of negative effects [29]. However, it seems that mycotoxin levels and duration of exposure may induce different effects on the antioxidant system. According to literature [29], most of the studies on this topic are based on aflatoxins (AFB1), which is reported to induce downregulation of antioxidant enzymes—such as superoxide dismutase, glutathione peroxidase, and catalase—thus resulting in increased lipid peroxidation by-products and a strong decrease in the levels of the predominant exogenous antioxidant compounds, such as glutathione. Accordingly, in our survey, we found a significant up-accumulation of oxidized glutathione (VIP score = 1.25) in milk samples belonging to cluster 3 (i.e., contamination by high levels of Aspergillus-mycotoxins), although no aflatoxins were detected in any corn silage samples [9]. Glutathione is an antioxidant molecule that helps protecting cells from reactive oxygen species such as free radicals and peroxides. By acting as an electron donor, glutathione reduces any disulfide bond formed within cytoplasmic proteins to cysteines. In this process, glutathione is converted to its oxidized form. In fact, the ratio of reduced glutathione to oxidized glutathione within cells is often used as a measure of cellular toxicity. Interestingly, corn silages belonging to cluster 3 were found to be highly contaminated by rugulusovin and brevianamide F [10]; therefore, as their toxic effect is mostly unknown in livestock but their presence in corn silages is notable, the significant accumulation of oxidized glutathione in milk samples belonging to the same cluster could be an indirect indicator of the oxidative imbalance caused by these *Aspergillus* toxins. However, as reported by Gallo et al. [10], future studies are needed to clarify the actual risk of their ingestion by farm animals and their impact on milk quality and production.

Our findings also showed that the different milk clusters differed when considering the metabolism of microbial nitrogen, with special reference to nucleic acids. In this regard, it is known that there is a direct positive relationship between microbial nucleic acids entering the small intestines and purine derivatives found in urine and milk [30,31]. The activity of xanthine oxidase, which catalyzed xanthine and hypoxanthine to uric acid, is very important in ruminant nutrition [32]. It provides the substrate pool (i.e., purine compounds) used in the salvage pathway for nucleic acids synthesis in the animal body tissues. Also, most of the dietary nucleic acids are mostly metabolized in the rumen and most of the microbial nucleic acids re-synthesized in the rumen flow into the lower gut where they are digested. It is very well known that milk contains some metabolites of purine and pyrimidine resulting from the microbial activity in the rumen of dairy cows [16,31]. The major purine metabolites are allantoin and uric acid, while hypoxanthine and xanthine are found only in small amounts. In this regard, the secretion of allantoin into milk seems to be through diffusion from plasma to the mammary alveolar lumen. Uric acid secreted into milk seems to be derived from plasma, and from the metabolism of endogenous purines in the mammary gland [31]. In our experimental conditions, intriguing results were observed when considering the average LogFC pairwise comparison of milk from high-contaminated vs. low-contaminated corn silage groups. Allantoin (structurally confirmed by MSMS approach) showed non-significant variations in the different milk samples, while uric acid (structurally confirmed by MSMS approach as well) was characterized by significant variations as function of the corn silages’ contamination (Table S1). More specifically, uric acid showed a significant down-accumulation in milk samples belonging to clusters 3 and cluster 5, being on average −0.86 and −3.22, respectively. Interestingly, the cluster 4 (i.e., milk samples from dairy cows fed with a corn silage contaminated by high levels of *Fusarium*-produced mycotoxins) showed a strong up-accumulation of uric acid when compared to the low-contaminated clusters, being the averaged LogFC value = 1.45. According to the degradation pathway of purine metabolism reported in KEGG, hypoxanthine is oxidized to xanthine, which is further oxidized to uric acid by xanthine oxidase. Accordingly, we found significant down-accumulation values for both adenosine and hypoxanthine in milk samples belonging to cluster 4. Therefore, we can speculate that purine nucleotides metabolism was one of the main pathways that changed according to the intake of corn contaminated corn silages. In a previous work, Wang et al. [15] identified nine milk metabolites that were significantly affected by the AFB1 contamination in their basal total mixed ration. In particular, the authors reported that the most affected metabolisms were those of phenylalanine, tyrosine, and tryptophan biosynthesis, phenylalanine metabolism, and arginine and proline metabolism. Therefore, these authors confirmed that the class represented by amino acids and peptides was particularly affected following the contamination by AFB1. In our experimental conditions, when considering the arginine and proline metabolism, a significant up-accumulation of spermidine for the clusters 3, 4, and 5 was found (Table S1). Milk assigned to cluster 5 (i.e., cows fed with silages contaminated by high levels of fumonisins and their metabolites) showed the higher up-accumulation values, recording an averaged LogFC = 4.1, followed by cluster 3 (averaged LogFC = 1.73) and cluster 4 (averaged LogFC = 0.74).

Finally, regarding the class of sphingolipids, 15 metabolites showed significant average LogFC variations when comparing the high-contaminated clusters with the low-contaminated ones (Table S1). Interestingly, some sphingolipids showed specific variations in cluster 5 (i.e., cows fed with silages contaminated by high levels of fumonisins and their metabolites). This trend is not novel; in this regard, it is known that fumonisins are structurally like sphingoid bases of sphinganine and sphingosine by possessing an unsubstituted primary amino group at C2 which competitively inhibits ceramide synthase thus disrupting the synthesis of ceramide and affecting the metabolism of sphingolipids. Also, a previous work [33] demonstrated that feed contaminated with regular levels of Fusarium mycotoxins adversely affected the metabolic profile of dairy cows. Therefore, the alterations of some sphingolipids and ceramides observed in this work could be related to an impaired sphingolipids metabolism, as previously reviewed by Loh et al. [9].

## 3. Materials and Methods

### 3.1. Collection of Milk Samples

In this work, 45 bulk milk samples were collected from January to June 2018 in dairy farms located in the Po Valley (Italy). These latter farmed Holstein-Friesian housed in free-stall barns without pasture access. The herds were characterized by having on average the 38.3% ± 1.9 of primiparous on lactating dairy cows and the 2.4 ± 0.2 lactations before culling. The dairy cows were milked twice a day (i.e., morning and afternoon milkings), fed a diet based on the large use of corn silage. The lactating dairy cows were fed with the same corn silages previously analysed by Gallo et al. [10] from at least four weeks, thus avoiding to collect milk in the period in which corn silage bunkers were changed. During the day of visit, we collected also information regarding herd composition, milk yield of lactating groups and milk quality, dry matter intake (DMI) as well as TMR formulation characteristics. The other recovered information is descriptively presented on Table 1, as average values for all cohort of visited dairy farms. The corn silage samples were collected at least 10 weeks after ensiling from horizontal bunker silos and their mycotoxin contamination profiles (i.e., regulated and emerging mycotoxins, with the latter presented as sum associated to specific fungal species within the four main mycotoxigenic genera of Aspergillus, Alternaria, Fusarium, and Penicillium spp.) are reported in Table 2, thus summarizing the previous results published by Gallo et al. [10]. A sample of bulk tank milk (500 mL) was collected on the same day of the visit. The corn silages were firstly divided in five clusters according to the mycotoxin contamination profiles, as reported in our previous work [10], namely: cluster 1 (corn silages contaminated by low levels of both Aspergillus- and Penicillium-produced mycotoxins); cluster 2 (corn silages contaminated by low levels of fumonisins, and other Fusarium-produced mycotoxins); cluster 3 (corn silages contaminated by high levels of Aspergillus-mycotoxins); cluster 4 (corn silages contaminated by high levels of Fusarium-produced mycotoxins); cluster 5 (corn silages contaminated by high levels of fumonisins and their metabolites; number of samples: 3). The collected 45 bulk milk samples were then classified according to the corn silages clusters previously reported, thus obtaining the following groups: 18 samples (cluster 1), 17 samples (cluster 2), 2 samples (cluster 3), 5 samples (cluster 4), and 3 samples (cluster 5) (Table S1).

### 3.2. Extraction of Milk Metabolites

The extraction of milk metabolites was carried out as previously reported in different works dealing with milk metabolomics [21,34,35]. Briefly, the 45 milk samples were skimmed by centrifugation at 4500× *g* for 10 min at 4 ℃, and then frozen at −80 ℃ for further processing. Thereafter, the milk samples were thawed and thoroughly vortex mixed. An aliquot of 2 mL of each sample was added to 14 mL of acetonitrile (LC-MS grade, Sigma-Aldrich, Madison, CA, USA) acidified with 3% formic acid, mixed by vortexing for 2 min and processed with ultrasounds for 5 min. Next, the samples were centrifuged at 12,000× *g* for 15 min at 4 ℃ to remove large biomolecules (such as proteins). The supernatants were then filtered through 0.22-μm cellulose syringe filters in amber vials until the further metabolomic analysis.

### 3.3. Untargeted Metabolomics Analysis

The untargeted UHPLC-HRMS analysis was performed on a Q Exactive™ Focus Hybrid Quadrupole-Orbitrap Mass Spectrometer (Thermo Scientific, Waltham, MA, USA) coupled to a Vanquish ultra-high-pressure liquid chromatography (UHPLC) pump and equipped with a heated electrospray ionization (HESI)-II probe (Thermo Scientific, USA). The chromatographic separation was achieved under a water-acetonitrile (both LC-MS grade, from Sigma-Aldrich, Milan, Italy) gradient elution (6–94% acetonitrile in 35 min) using 0.1% formic acid as phase modifier, on an Agilent Zorbax Eclipse Plus C18 column (50 × 2.1 mm, 1.8 μm). The HRMS conditions were adapted according to previously optimized conditions based on Orbitrap MS-analyzers [36]. Briefly, the flow rate was 200 μL/min and for the full scan MS analysis, a positive ionization mode with a mass resolution of 70,000 at *m*/*z* 200 was used. The injection volume was 6 μL, using a *m*/*z* range of 70–1200. The automatic gain control target (AGC target) and the maximum injection time (IT) were 1 × 10^6^ and 200 ms, respectively. Randomized injections of pooled quality control (QC) samples were acquired in a data-dependent (Top N = 3) MS/MS mode with full scan mass resolution reduced to 17,500 at *m*/*z* 200, with an AGC target value of 1 × 10^5^, maximum IT of 100 ms, and isolation window of 1.0 *m*/*z*, respectively. The Top N ions were selected for fragmentation under stepped Normalized Collisional Energy (i.e., 10, 20, 40 eV). The HESI parameters for both MS and MS/MS were as follows: sheath gas flow 40 arb (arbitrary units), auxiliary gas flow 20 arb, spray voltage 3.5 kV, capillary temperature 320 °C. Prior to data collection, the mass spectrometer was calibrated using Pierce™ positive ion calibration solution (Thermo Fisher Scientific, San Jose, CA, USA). To avoid possible bias, the sequence of injections was randomized. The collected data (.RAW files) were converted into abf format using the Reifycs Abf Converter and then further processed using the software MS-DIAL (version 4.60) [37]. Automatic peak finding, LOWESS normalization, and annotation via spectral matching against the database Bovine Metabolome Database (BMDB) [23] were performed. The mass range 100–1200 *m*/*z* was searched for features with a minimum peak height of 10,000 cps. The MS and MS/MS tolerance for peak centroiding was set to 0.01 and 0.05 Da, respectively. Retention time information was excluded from the calculation of the total score. Accurate mass tolerance for identification was 0.01 Da for MS and 0.05 Da for MS/MS. The identification step was based on mass accuracy, isotopic pattern, and spectral matching. In MS-Dial, these criteria were used to calculate a total identification score. The total identification score cut off was >50%, considering the most common HESI+ adducts. Gap filling using peak finder algorithm was performed to fill in missing peaks, considering 5 ppm tolerance for *m*/*z* values. The software MS-Finder [38] was used for in-silico fragmentation of the non-annotated mass features, using Lipid Maps, FoodDB, and BMDB libraries, thus reaching a level 2 of confidence in annotation [22]. In particular, those compounds presenting an in-silico prediction score > 5 were retained.

### 3.4. Multivariate Statistics and Pathway Analysis

The multivariate statistical elaboration of metabolomics-based data was performed using three different tools, namely Mass Profiler Professional (from Agilent Technologies, Santa Clara, CA, USA), MetaboAnalyst and SIMCA 13 (Umetrics, Malmo, Sweden), as reported in previous works [21,34]. Briefly, after data normalization, both unsupervised and supervised multivariate statistics were carried out. In this regard, the unsupervised approach was based on hierarchical cluster analysis (HCA) and principal component analysis (PCA), while the orthogonal projections to latent structures discriminant analysis (OPLS-DA) was used as supervised tool. Additionally, the OPLS-DA model validation parameters (goodness-of-fit R^2^Y together with goodness-of-prediction Q^2^Y) were inspected, considering a Q^2^Y prediction ability of >0.5 as the acceptability threshold. Thereafter, the OPLS-DA model produced was inspected for outliers and permutation testing (N > 100) was performed to exclude model over-fitting. The importance of each milk metabolite for discrimination purposes (i.e., when considering the different clusters as related to the silages contamination) was then calculated according to the variable selection method VIP (i.e., variables importance in projection), considering as the minimum significant threshold those values higher than 0.8 [39]. As the next step, volcano plots were produced for the comparison between contaminated (cluster 3, 4, and 5) vs. control groups (cluster 1 and 2) by coupling fold-change analysis (cut-off value > 1.2) and ANOVA (*p* < 0.05; post hoc test: Tukey HSD; multiple testing correction: Bonferroni Family-Wise Error Rate). Finally, the online tool MetaboAnalyst was used to inspect both the most important pathways represented by the metabolites annotated (using as pathway library: Bos taurus, KEGG), and then to provide a metabolite set enrichment analysis based on the discriminant and significant class of metabolites outlined by both Volcano plots and VIP selection method.

## 4. Conclusions

In this research survey, milk metabolomics was found to be a potential tool to discriminate mycotoxin patterns in contaminated corn silages. However, the accuracy of the prediction is still not robust enough and further ad-hoc and targeted studies are required to confirm the robustness of the milk biomarkers proposed. Also, this survey represents an observational study, revealing an association between certain mycotoxins-contaminating corn silages sampled on farm and milk metabolite profiles in bulk milk of dairy cows. Additionally, one of the main reasons behind the difficulty of using milk metabolomics to reveal indirect contamination of corn silages since the mammary gland is a dynamic organ but distal from the rumen, where most of the microbial processing and detoxification of mycotoxins occur when considering dairy cows. Additionally, the presence of other intermediate organs, like the liver and the intravascular blood compartment, contribute to further modulate the milk metabolome and this represents another aspect should be better assessed in future studies to verify how mycotoxins from silage could interfere with immune-metabolic profile of animals. Nevertheless, in this work, we have highlighted the most affected chemical classes of milk metabolites changing by ingestion of not only regulated—but also emerging—mycotoxins into corn silage, thus providing potential milk biomarkers. However, in future works, the impact of other potential sources of mycotoxins in the feeding ratios and considering individual feeding animals must be deepened. Besides, future works should measure the intake of corn silage (not as a field estimation), considering that the proportion of corn silage in the diet can vary across time due to changes in DM content of the material, which changes overtime, and mixing or weighing errors in the dairy farm. In fact, to confirm causal relationship of mycotoxin pattern on milk metabolome the appropriate experimental approach should be based on controlled and standardized feeding of contaminated silages to cows and studying the individual change of milk metabolome.

## Figures and Tables

**Table 1:** Descriptive statistics of herd composition and characteristics, together with parameters related to milk yield and quality, dry matter intake, dietary composition, and ingredient inclusion.

Items	Unit	Mean	S.D.	Q_25_	Q_75_
Herd composition and characteristics					
Lactating cows	n	199.7	138.2	94.0	270.0
Dry cows	n	27.9	25.8	10.0	40.0
Calves	n	193.6	140.4	94.0	264.0
Milk yield and quality					
Milk yield	Kg/cow/day	32.3	4.6	29.8	35.2
4% Fat corrected milk	Kg/cow/day	33.40	4.92	30.16	36.30
Energy corrected milk	Kg/cow/day	35.50	5.14	32.23	38.75
Milk fat	%	4.03	0.17	3.94	4.13
Milk protein	%	3.50	0.10	3.44	3.55
Milk casein	%	2.72	0.09	2.68	2.77
SCC	log_10_ cells/mL × 1000	5.23	0.16	5.11	5.34
Dry matter intake, dietary composition ^a^ and ingredient inclusion					
Dry matter intake	Kg DM/cow/day	23.76	2.24	22.79	25.49
Net energy for lactation _3x_^b^	Mcal/kg DM	1.63	0.04	1.61	1.66
CP	% DM	16.42	1.04	15.75	17.19
Soluble CP	% DM	4.77	0.37	4.57	5.02
NDF	% DM	29.85	3.26	27.65	31.38
ADF	% DM	19.12	2.43	17.71	20.16
NFC	% DM	41.67	2.55	39.55	43.62
Starch	% DM	27.46	3.41	25.99	29.78
Corn silage ^c^	%	30.51	5.84	25.98	35.64

SCC = somatic cell count; CP = crude protein; CSS: NDF = neutral detergent fiber; ADF = acid detergent fiber; NFC = Non-Fiber Carbohydrate. ^a^ As calculated by formulating diet with NRC (2001) animal nutritional model. ^b^ Net Energy for lactation calculated at 3 times the maintenance (NRC, 2001). ^c^ Inclusion level of specific ingredients calculated on the total dietary dry matter.

**Table 2:** Descriptive statistics for dry matter, fermentative traits, and mycotoxins concentrations of corn silage belonging to the five different clusters. For more details see Gallo et al. [10].

		Cluster				
Items	Units	1	2	3	4	5
Dry matter	%	34.5	34.4	37.9	37.4	36.2
pH		3.69	3.77	3.84	3.80	3.89
1,2-propanediol	% DM	0.57	0.42	0.73	0.51	0.19
Acetic acid	% DM	3.38	3.24	3.28	2.87	3.03
Propionic acid	% DM	0.12	0.16	0.04	0.07	0.25
Butyric acid	% DM	0.001	0.003	0.001	0.003	0.003
Lactic acid	% DM	3.33	4.29	1.91	3.47	2.51
Mycotoxins concentrations ^a^ and incidence						
Zearalenone	µg/kg DM	4.61 ± 2.93	28.84 ± 45.20	7.11 ± 4.05	2.93 ± 1.69	1.63 ± 0.34
	Incidence	33.3%	18.8%	100.0%	20.0%	66.7%
Deoxynivalenol	µg/kg DM	43.65 ± 46.79	25.59 ± 39.08	151.67 ± 8.89	32.33 ± 26.77	96.87 ± 130.93
	Incidence	61.1%	68.8%	100.0%	100%	66.7%
Fumonisin B1	µg/kg DM	105.72 ± 110.69	210.10 ± 214.76	181.80 ± 123.04	268.15 ± 131.28	1576.64 ± 265.53
	Incidence	94.4%	100%	100%	100%	100%
Fumonisin B2	µg/kg DM	27.37 ± 33.70	68.49 ± 83.85	139.13 ± 187.23	69.90 ± 17.84	404.73 ± 50.32
	Incidence	94.4%	93.8%	100%	100%	100%
Fumonisin B3	µg/kg DM	11.40 ± 8.47	18.72 ± 16.69	69.00 ± 59.72	24.97 ± 6.05	232.54 ± 120.66
	Incidence	66.7%	100%	100%	100%	100%
Moniliformin	µg/kg DM	12.47 ± 13.36	7.12 ± 7.73	39.90 ± 5.61	12.97 ± 9.04	36.22 ± 34.38
	Incidence	94.4%	93.8%	100%	100%	100%
Fusaric acid	µg/kg DM	165.93 ± 92.69	210.62 ± 105.41	195.48 ± 88.71	209.49 ± 174.44	375.11 ± 408.74
	Incidence	100%	100%	100%	100%	100%
Sum of *Aspergillus* toxins	µg/kg DM	161.52 ± 143.74	93.09 ± 62.34	565.23 ± 230.06	46.40 ± 33.42	236.22 ± 190.16
	Incidence	100%	100%	100%	100%	100%
Sum of *Alternaria* toxins	µg/kg DM	7.71 ± 9.86	1.35 ± 4.28	18.65 ± 13.87	3.65 ± 8.15	38.39 ± 29.49
	Incidence	100%	100%	100%	100%	100%
Sum of *Fusarium* ^b^ toxins	µg/kg DM	230.19 ± 99.40	716.42 ± 117.73	619.71 ± 99.76	1567.17 ± 340.46	739.80 ± 445.34
	Incidence	100%	100%	100%	100%	100%
Sum of *Penicillium* toxins	µg/kg DM	162.23 ± 138.24	96.78 ± 93.55	708.24 ± 116.77	80.51 ± 41.46	189.66 ± 139.68
	Incidence	100%	100%	100%	100%	100%

^a^ Concentrations represented the average values of only detectable mycotoxins. ^b^ The sum of *Fusarium* toxins does not include the singular mycotoxins (such as fumonisins) reported above.

**Table 3:** Pairwise comparisons of milk samples from contaminated (i.e., cluster 3, 4, and 5) and low-contaminated corn silage clusters (i.e., cluster 1 and 2). The variations of the main classes are expressed as average Log2 fold-change (FC) values. The most discriminant compounds according to the OPLS-DA prediction model built (VIP score > 1) together with their adjusted *p*-values (resulting from Volcano plot analysis and family-wise error rate) are also reported.

Superclass	Most Discriminant Compound	LogFC Cluster 3 vs. Cluster 1	LogFC Cluster 3 vs. Cluster 2	LogFC Cluster 4 vs. Cluster 1	LogFC Cluster 4 vs. Cluster 2	LogFC Cluster 5 vs. Cluster 1	LogFC Cluster 5 vs. Cluster 2
Alkaloids and derivatives	Ecgonine (VIP score: 1.54; *p*-value: 2.55 × 10^−4^)	0.061	−0.020	0.526	0.445	−1.031	−1.112
Amines	Norspermidine (VIP score:1.57; *p*-value: 2.82 × 10^−5^)	−0.342	−0.232	0.052	0.162	0.583	0.692
Amino acids and peptides	*O*-Succinyl-*L*-homoserine (VIP score: 1.78; *p*-value: 5.36 × 10^−5^)	0.144	0.325	−0.199	-0.018	0.775	0.956
Benzenoids	1-Hydroxypyrene glucuronide (VIP score: 1.74; *p*-value: 2.25 × 10^−4^)	0.139	0.198	−0.278	−0.219	0.447	0.507
Diazines	Aripiprazole (VIP score: 1.49; *p*-value: 1.93 × 10^−3^)	−0.227	−0.127	−0.759	−0.659	0.989	1.089
Fatty Acyls	Nephritogenoside (VIP score: 1.29; *p*-value: 2.83 × 10^−5^)	−0.200	−0.174	−0.160	−0.135	−0.119	−0.093
Glycerolipids	TG(15:0/24:0/24:1(15Z)) (VIP score: 1.40; *p*-value: 1.67 × 10^−10^)	0.528	0.435	0.297	0.204	0.728	0.635
Glycerophospholipids	DG(18:0/22:6(4Z,7Z,10Z,13Z,16Z,19Z)) (VIP score: 1.42; *p*-value: 1.67 × 10^−12^)	0.172	0.088	0.191	0.107	−0.0002	−0.085
Indoles and derivatives	Indoleacetic acid (VIP score: 1.79; *p*-value: 4.49 × 10^−5^)	0.043	0.156	0.135	0.249	0.169	0.282
Keto acids and derivatives	4-Fumarylacetoacetic acid (VIP score: 1.03; *p*-value: 1.01 × 10^−2^)	−0.512	−0.268	0.105	0.349	0.475	0.718
Nucleosides, nucleotides, and analogues	8-Oxo-dGMP (VIP score: 1.19; *p*-value: 3.69 × 10^−5^)	−0.459	−0.496	0.673	0.635	−1.556	−1.593
Organic acids and derivatives	Taurine (VIP score: 1.25; *p*-value: 6.19 × 10^−7^)	1.130	1.437	−0.152	0.1544	1.817	2.124
Organooxygen compounds	Chitobiose (VIP score: 1.97; *p*-value: 2.08 × 10^−3^)	0.810	0.767	0.134	0.092	0.696	0.654
Polyphenols	Troxerutin (VIP score: 1.42; *p*-value: 5.69 × 10^−10^)	0.010	0.389	−0.223	0.155	0.044	0.423
Prenol lipids	Farnesol (VIP score: 1.26; *p*-value: 1.19 × 10^−2^)	−0.244	−0.348	0.573	0.468	−1.260	−1.365
Pteridines and derivatives	Pteroyl-D-glutamic acid (VIP score: 1.21; *p*-value: 2.08 × 10^−4^)	0.734	0.900	−0.214	−0.048	1.546	1.712
Purine nucleotides	ADP-ribose 2′-phosphate (VIP score: 1.31; *p*-value: 1.19 × 10^−5^)	1.496	2.329	−0.332	0.501	2.810	3.644
Purine nucleosides	Guanosine (VIP score: 1.34; *p*-value: 2.82 × 10^−4^)	0.820	0.696	0.987	0.863	0.426	0.302
Pyridines and derivatives	N-Methylnicotinium (VIP score: 0.99; *p*-value: 8.94 × 10^−5^)	−0.454	−0.961	−0.070	−0.576	−1.718	−2.225
Pyrimidine nucleosides	Thymidine (VIP score: 1.09; *p*-value: 5.41 × 10^−3^)	−0.602	−0.886	−0.738	−1.022	−0.198	−0.481
Pyrimidine nucleotides	dUMP (VIP score: 1.29; *p*-value: 2.93 × 10^−6^)	1.496	2.329	−0.332	0.501	2.810	3.644
Imidazopyrimidines	Hypoxanthine (VIP score: 1.04; *p*-value: 1.61 × 10^−3^)	−0.480	-0.409	0.054	0.125	−0.039	0.031
Quinolines and derivatives	Salsoline-1-carboxylate (VIP score: 1.05; *p*-value: 2.98 × 10^−3^)	−0.317	−0.237	−0.594	−0.515	0.068	0.148
Sphingolipids	Phosphatidylinositol-3,4,5-trisphosphate (VIP score: 1.34; *p*-value: 1.47 × 10^−3^)	0.552	0.595	0.362	0.405	0.587	0.630
Steroids and steroid derivatives	Vitamin D2 3-glucuronide (VIP score: 1.42; *p*-value: 1.45 × 10^−13^)	−0.135	0.120	−0.179	0.077	0.554	0.809
Tetrapyrroles and derivatives	Mesoporphyrin IX (VIP score: 1.55; *p*-value: 1.33 × 10^−15^)	0.859	1.184	−0.578	−0.254	2.706	3.031
Other metabolites	Choline (VIP score: 1.25; *p*-value: 2.12 × 10^−4^)	−0.711	−0.219	−0.748	−0.256	0.205	0.697

## Data Availability

The Supplementary Material will be also publicly available as Mendeley Data on the reserved doi:10.17632/hv9dskxhmz.2.

## Supplementary Materials

The following are available online at https://www.mdpi.com/article/10.3390/metabo11080475/s1, Figure S1: Metabolomic view map according to metabolites identified in milk from dairy cows fed with mycotoxins-contaminated corn silages. The *x*-axis represents the pathway impact, and *y*-axis represents the pathway enrichment. Larger sizes and darker colors represent higher pathway enrichment and higher pathway impact values, respectively, Table S1: Metabolomic dataset resulting from the UHPLC-HRMS analysis containing all the annotated metabolites, together with their level of confidence in annotation, relative abundance, and composite mass spectrum (MS and MSMS transitions). The discriminant milk metabolites passing the different statistical approaches (i.e., Volcano Plots and VIP selection method) together with the cross-validation parameters of the OPLS-DA prediction model built and the PCA score plot are also provided.
