# Supplementary material for: Changes of Milk Metabolomic Profiles Resulting from a Mycotoxins-Contaminated Corn Silage Intake by Dairy Cows
Source: Metabolites. 2021 Jul 23;11(8):475. doi: 10.3390/metabo11080475 (PMC8400352; doi:10.3390/metabo11080475)

Supplementary Figure S1. Metabolomic view map according to metabolites identified in milk from dairy cows fed with mycotoxins-contaminated corn silages. The x-axis represents the pathway impact, and y-axis represents the pathway enrichment. Larger sizes and darker colors represent higher pathway enrichment and higher pathway impact values, respectively.

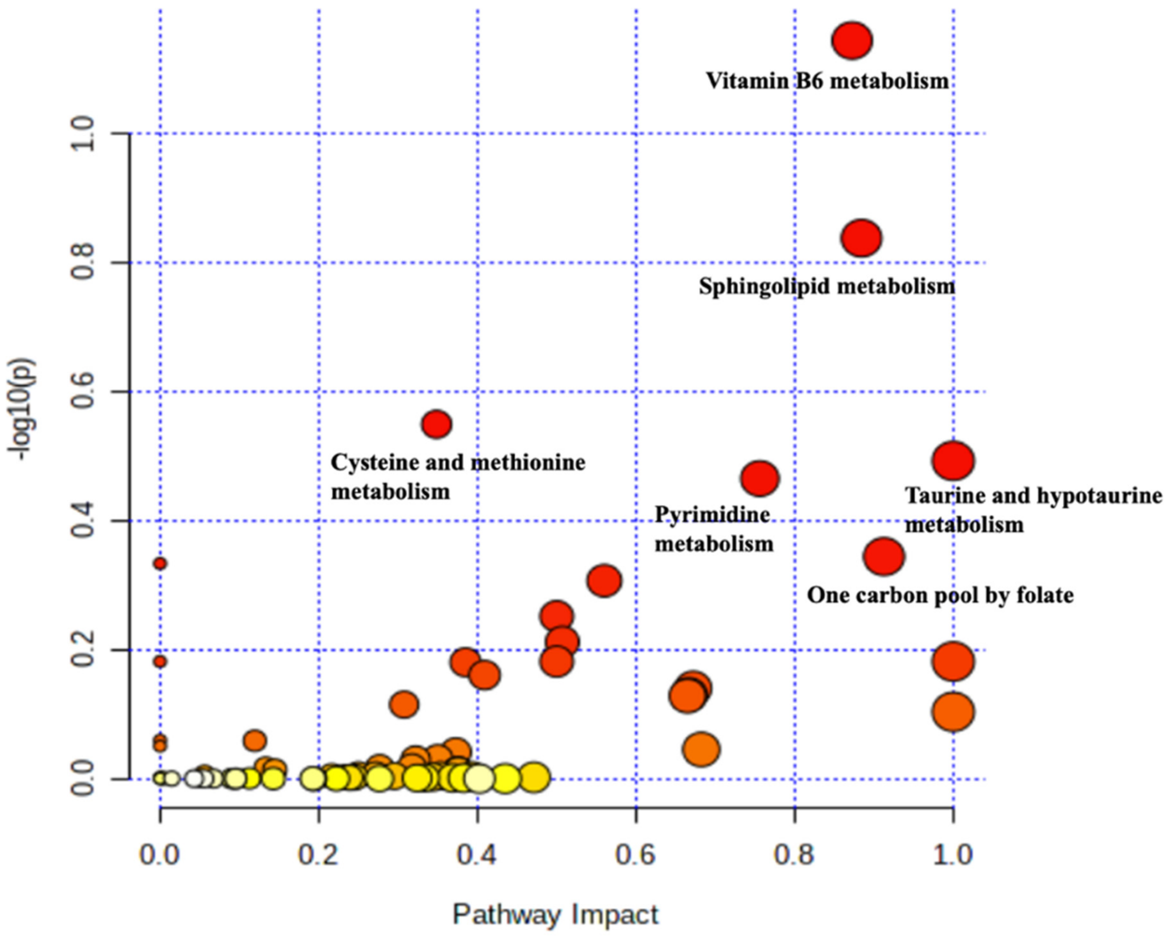

Supplement: Supplementary file 1 [file metabolites-11-00475-s001.zip › Figure S1.pdf]
